# LncRNA *SNHG1* regulates neuroblastoma cell fate via interactions with HDAC1/2

**DOI:** 10.1038/s41419-022-05256-z

**Published:** 2022-09-21

**Authors:** Chia-Lang Hsu, Chieh-Fan Yin, Yi-Wen Chang, Ya-Chih Fan, Shih-Han Lin, Yu-Ching Wu, Hsuan-Cheng Huang, Hsueh-Fen Juan

**Affiliations:** 1 Department of Medical Research, National Taiwan University Hospital, Taipei, 100225 Taiwan; 2 Graduate Institute of Oncology, National Taiwan University College of Medicine, Taipei, 100225 Taiwan; 3 Graduate Institute of Medical Genomics and Proteomics, National Taiwan University College of Medicine, Taipei, 100225 Taiwan; 4 Center for Computational and Systems Biology, National Taiwan University, Taipei, 10617 Taiwan; 5 Department of Life Science and Institute of Molecular and Cellular Biology, National Taiwan University, Taipei, 10617 Taiwan; 6 Institute of Biomedical Informatics, National Yang Ming Chao Tung University, Taipei, 112 Taiwan; 7 Graduate Institute of Biomedical Electronics and Bioinformatics, National Taiwan University, Taipei, 10617 Taiwan

**Keywords:** RNA sequencing, Cancer genetics, Long non-coding RNAs

## Abstract

The small nucleolar RNA host gene 1 (*SNHG1*) is a novel oncogenic long non-coding RNA (lncRNA) aberrantly expressed in different tumor types. We previously found highly expressed *SNHG1* was associated with poor prognosis and *MYCN* status in neuroblastoma (NB). However, the molecular mechanisms of *SNHG1* in NB are still unclear. Here, we disrupted endogenous *SNHG1* in the *MYCN*-amplified NB cell line SK-N-BE(2)C using the CRISPR/Cas9 system and demonstrated the proliferation and colony formation ability of *SNHG1*-knowndown cells were suppressed. The transcriptome analysis and functional assays of *SNHG1*-knockdown cells revealed *SNHG1* was involved in various biological processes including cell growth, migration, apoptosis, cell cycle, and reactive oxygen species (ROS). Interestingly, the expression of core regulatory circuitry (CRC) transcription factors in *MYCN*-amplified NB, including *PHOX2B, HAND2, GATA3, ISL1, TBX1*, and *MYCN*, were decreased in *SNHG1*-knockdown cells. The chromatin-immunoprecipitation sequencing (ChIP-seq) and transposase-accessible chromatin using sequencing (ATAC-seq) analyses showed that chromatin status of these CRC members was altered, which might stem from interactions between *SNHG1* and HDAC1/2. These findings demonstrate that *SNHG1* plays a crucial role in maintaining NB identity via chromatin regulation and reveal the function of the lncRNA *SNHG1* in NB.

## Introduction

Neuroblastoma (NB) is the most common solid tumor that occurs in the developing sympathetic nervous system during childhood [1]. NB arises at a frequency of approximately 10.3 per million for children under 15 years old [2]. What causes NB is still unclear, but it is related to defects in how neural crest cells migrate from the dorsal neural tube or differentiate upon reaching their apposite locations into organs and tissues of the sympathetic nervous system [3]. NB commonly begins in one of the adrenal glands, but can also develop in the chest, neck, abdomen, spine, or brain. It quickly expands to other parts of the body and metastasizes in 50 to 60% of all cases. Several genomic alterations have been identified in NBs, and the most common genetic alteration is the amplification of the oncogene *MYCN* [4]. *MYCN* amplification is observed in about 25% of NB cases [5] and highly correlated with rapid tumor progression and poor clinical outcome in patients of all ages [6].

Over the past decades, a great deal of evidence shows that the non-coding portion of the genome, such as microRNAs and long non-coding RNAs (lncRANs), plays a fundamental role in many cancers. LncRNAs are RNA transcripts longer than 200 nucleotides and without protein coding region [7, 8] and have been demonstrate they are correlated with a variety of developmental processes and human diseases by involving in chromosome remodeling mechanisms, transcriptional gene regulation, and post-transcriptional processing [9]. LncRNAs can interact with an extensive range of highly specific substrates to regulate and modulate the gene expression [10]. Many lncRNAs have been proposed to be associated with NB [11].

Small nucleolar RNA host gene 1 (*SNHG1)* is identified as one kind of lncRNA and involved in several cancers’ regulation, such as colon cancer, hepatocellular carcinoma, and NB [12]. In particular, it is associated with various carcinogenesis processes, but its role in carcinogenesis is different according to the various types of tumors. Our previous study used a bioinformatic approach to identify a set of *MYCN*-status-associated lncRNAs [13]. We further demonstrated that a higher expression level of *SNHG1* was significantly associated with worse outcomes in NB and can be a prognostic marker for predicting the clinical outcome of NB patients [13]. We also identified a set of *SNHG1*-interacting proteins and proposed that *SNHG1* enhances NB progression by interacting with MATR3 to influence splicing events [4]. A previous work showed that *SNHG1* conduces to tumorigenesis in NB by acting as a sponge for miR-338-3p to regulate PLK4 expression [14]. In this study, we found a novel molecular mechanism of the role of *SNHG1* in NB. These results provide useful insights and may be a valuable resource for NB therapeutics.

## Materials and methods

### Cell culture

The human NB cell line SK-N-BE(2)C was obtained from the America Type Tissue Collection (ATCC; Manassas, VA, USA). The cell line was cultured in Dulbecco’s Modified Eagle’s Medium (DMEM; Thermo Fisher Scientific, Waltham, MA, USA) with 10% fetal bovine serum (FBS; Thermo Fisher Scientific) and 1.5 g/L sodium bicarbonate (Sigma-Aldrich, St. Louis, MO, USA). The cells were grown at 37 °C in a 5% carbon dioxide atmosphere. SK-N-BE(2)C cell line was authenticated by STR profiling and tested for mycoplasma contamination.

### CRISPR/Cas9 depletion of *SNHG1* expression in SK-N-BE(2)C cells

We used an all-in-one sgRNA/Cas9 system, which can simultaneously express Cas9 proteins and sgRNA. The sgRNA was designed using the CRISPR Design Tool (http://crispr.mit.edu/). The sgRNA sequence with the highest score for targeting *SNHG1* was cloned into the all-in-one system sgRNA/Cas9 expression vector (pAll-Cas9.Ppuro). We used pSurrogate reporters that contained the sgRNA-targeting site and enhanced green fluorescent protein (EGFP) and mCherry fluorescent protein sequences. sgRNA information: 5’-CAG ATG TCC CTT CAA GTT CC-3’ (one region around *SNHG1* genome locus) and 5’-CTG AAC TTG CGG CGC GAA AA-3’ (another region around *SNHG1* genome locus). These customized plasmids were purchased from the National RNAi Core Facility (NRC; Academia Sinica, Taipei, Taiwan). On the day of transfection, cells were optimal at 80% confluence. Constructs carrying the sgRNA/Cas9 and pSurrogate reporters were introduced into cells by co-transfection using Lipofectamine 3000 transfection reagent (Thermo Fisher Scientific), according to the manufacturer’s protocol. At 24 h post-transfection, the culture medium was replaced with fresh medium containing 1 μg/mL puromycin (Sigma-Aldrich) to remove non-transfected cells. Cells were incubated for a total of 48 h post-transfection. After 7 days of puromycin selection, single-cell clones were isolated by fluorescence cell sorting into 96-well plates and incubated for 2 weeks. The sorted *SNHG1*-depletion clones were then expanded in 12-well plates, followed by extraction of total RNA for qRT-PCR.

### Construction of the *SNHG1* overexpression plasmid

The *SNHG1* sequence was amplified from the cDNA of SK-N-BE(2)C by polymerase chain reaction (PCR) in a T100^TM^ Thermal Cycler (Bio-Rad, Hercules, CA, USA) and cloned into the pcDNA3.1(+) vector. The two sets of *SNHG1-*specific oligonucleotide primers were as follows: *SNHG1*-Foward-NotI 5’-CAG CAC AGT G GCGGCCGC CTC ATT TTT CTA CTG CTC G −3’ and *SNHG1*-Reverse-XhoI 5’-G CCC TCT AGA CTCGAG AAA CAG GAC TAT GTA ATC AAT −3’. The primers incorporated the NotI (Thermo Fisher Scientific) and XhoI (Thermo Fisher Scientific) restriction sites. The *SNHG1* overexpression plasmid was sequenced at the DNA Sequencing Facility (Genomics BioSci & Tech, Taipei, Taiwan). The *SNHG1* overexpression plasmid was transformed into the *Escherichia coli* (*E. coli*) strain DH5-alpha. Ampicillin selection was performed to select the colonies carrying *SNHG1* overexpression plasmids.

### Cell transfection

SK-N-BE(2)C cells were transfected for 48 h with a pcDNA3.1(+) control vector or pcDNA3.1-*SNHG1* expression vector using Lipofectamine 3000 (Thermo Fisher Scientific), according to the manufacturer’s protocol. Cells were harvested at 48 h post-transfection for further assays.

### RNA extraction and reverse transcription

We seeded *SNHG1*-depletion stable-clone (sgSNHG1) and control (sgCtrl) SK-N-BE(2)C cells in 10 cm dishes until they grew to 80–90% confluence. Next, 1 mL TRIzol® Reagent (Thermo Fisher Scientific) was added to the cells and the solution was homogenized. Cell lysates were collected and kept in room temperature for 5 min, after which 200 μL/mL chloroform (Sigma-Aldrich) was added and the lysate was vortexed for 15 s to extract cellular RNA. The solution was maintained at room temperature for 5 min and centrifuged at 12,000 × *g* at 4 °C for 20 min. The supernatant was collected into a new tube with an equivalent volume of 100% ethanol (Sigma-Aldrich), inverted, and kept at room temperature for 10 min. The mixture was then transferred into a Zymo-Spin IIC Column (Zymo Research, Irvine, CA, USA) and centrifuged at 13,000 × *g* at room temperature for 1 min. RNA wash buffer (400 μL) was added to the column, which was then centrifuged at 13,000 × *g* at room temperature for 1 min. DNase I (5 μL) and DNA Digestion Buffer (75 μL) were added into the column center and the column was centrifuged at 13,000 × *g* at room temperature for 1 min. Direct-zol RNA Prewash (400 μL) was added to the column, which was then centrifuged at 13,000 × *g* at room temperature for 1 min. The washing step was then repeated. RNA Wash Buffer (700 μL) was then added to the column, which was then centrifuged at 13,000 × *g* at room temperature for 4 min. Flow-through was discarded and the column was centrifuged at 13,000 × *g* for 4 min. The column was then transferred into a new RNase-free tube. RNase-free water (30 μL) was added into the column matrix, and the column was centrifuged at 13,000 × *g* for 1 min to elute the RNA. We then used a NanoDrop ND-1000 instrument (Thermo Fisher Scientific) to determine RNA concentration. The RNA quality was assessed with 1.5% gel electrophoresis. We reverse transcribed 1000 ng total RNA template of each sample to cDNA using a RevertAid^TM^ H Minus First Strand cDNA Synthesis Kit (Thermo Fisher Scientific) with oligo dT and random hexamer primers. Finally, the cDNA samples were stored at –20 °C until they were used.

### Real-time quantitative polymerase chain reaction (qRT-PCR)

The cDNA sample was amplified and assessed using a CFX Connect^TM^ Real-Time PCR Detection System (Bio-Rad). Each lncRNA expression value was measured using △△Ct and normalized to *GAPDH* or *HPRT*. All experiments were performed in triplicate. Primer information is in Table S1.

### Cell viability assay

We seeded 2.5 × 10^5^ cells of sgSNHG1 and sgCtrl SK-N-BE(2)C cells in each well of a 6-well plate. All cells were incubated in DMEM containing 10% FBS for 0, 24, 48, 72, 96, or 120 h. The cell viability was determined by a CellTiter 96^®^ Aqueous Non-Radioactive Cell Proliferation Assay (MTS; Promega, Mannheim, Germany). For each duration, cells were seeded at 5 × 10^3^ cells per well in a 96-well plate. We then added 20 μL of MTS/PMS (Promega) solution to each well and incubated for 1.5 h at 37 °C with 5% carbon dioxide. Absorbance was measured at 490 nm using a model 680 microplate reader (Bio-Rad).

### Colony formation assay

We seeded 1 × 10^3^ cells of sgSNHG1 and sgCtrl SK-N-BE(2)C cells in each well of a 6-well plate, and followed this by culturing in DMEM with 10% FBS for 10 days to assess colony formation. Colonies were fixed with methanol (Sigma-Aldrich) and stained with 0.5% crystal violet (Sigma-Aldrich). The numbers of colonies were quantified by counting.

### Cell apoptosis assessments

We seeded 5 × 10^6^ cells of sgSNHG1 and sgCtrl SK-N-BE(2)C cells in 15 cm dishes. All cells were incubated in DMEM containing 10% FBS for 72 h. For apoptosis detection, cells were washed with PBS, trypsinized, and stained with FITC-annexin V (BD Biosciences, Franklin Lakes, NJ, USA) and 0.5 mg/mL propidium iodide (PI; BD Biosciences), according to the manufacturer’s instructions. We acquired, measured, and analyzed 10,000 stained cells on a FACSCanto instrument (BD Biosciences).

### Cell cycle analysis

We seeded 2 × 10^6^ cells of sgSNHG1 and sgCtrl SK-N-BE(2)C cells in a 10 cm dish with DMEM containing 10% FBS for 24 h. Cells were then incubated in a serum-free medium. After 24 h incubation, cells were changed to DMEM containing 10% FBS. Cells were then washed with PBS, incubated with 10 mg/mL PI (BD Biosciences) for 1 h, and subjected to flow cytometry analysis (BD Biosciences). We collected 5000 cells for each measurement.

### Cell migration assay

We seeded 3 × 10^5^ cells of sgSNHG1 and sgCtrl SK-N-BE(2)C cells in each well of a 6-well plate. After 24 h incubation, cells were starved for 24 h in serum-free DMEM. We then reseeded 1 × 10^5^ cells with DMEM medium containing 1% FBS onto the upper chamber of an 8 µm pore size Transwell plate (Corning Incorporated, Corning, NY, USA). Cells were incubated at 37 °C with 5% carbon dioxide for 18 h. Cells were then fixed for 5 min with 3.7% paraformaldehyde (PFA; Sigma-Aldrich) in dark conditions, washed twice with PBS, then fixed in 100% methanol (Sigma-Aldrich) overnight in dark conditions. Cells were stained with 10% Giemsa stain (Sigma-Aldrich) for 1 day. Cotton swabs were used to remove cells from the upper side of the inserts. Images of five different microscope fields of each insert were captured and cell numbers were quantified by counting.

### Reaction oxygen species (ROS) assay

We seeded 2 × 10^6^ cells of sgSNHG1 and sgCtrl SK-N-BE(2)C cells in 10 cm dishes. All cells were incubated in DMEM containing 10% FBS for 24 h. Cells were then washed with PBS, trypsinized, and incubated with 1 mM 2’,7’-dichlorofluorescein diacetate (H_2_DCFDA; Thermo Fisher Scientific) in the dark at 37 °C for 30 min. Cells were then washed twice with PBS and analyzed using a FACSCanto instrument (BD Biosciences).

### Sodium butyrate (NaB) treatment

NaB powder (Sigma-Aldrich) was dissolved in double distilled water (ddH_2_O). Cells were treated with 1, 5, or 10 mM NaB. qRT-PCR and western blots were performed after 8 h.

### Western blots

We seeded cells from the sgSNGH1 and sgCtrl SK-N-BE(2)C cell line in 10 cm dishes until they grew to 80–90% confluence, after which protein lysates were extracted. Protein lysates were separated by molecular weight with electrophoresis through a 10% polyacrylamide gel, then transferred to 0.45 µm polyvinyl difluoride membranes (PVDF; Merck Millipore, Burlington, MA, USA). Blots were blocked for 1 h at room temperature with TBST (1x TBS, 0.1% Tween 20) containing 5% skim milk. The membrane was hybridized overnight with primary antibodies solubilized in 5% skim milk/TBST at 4 °C. The primary antibodies were as follows: anti-HDAC1 (ab7028, Abcam, Cambridge, UK), anti-HDAC2 (ab7029, Abcam), anti-Histone H3 (acetyl K27; ab4729, Abcam), anti-Histone H3 (GTX115549, Genetex, Irvine, CA, USA), and anti-Actin (MAB1501, Merck Millipore). Membranes were washed the following day with TBST three times for 5 min. The horseradish peroxidase-conjugated secondary antibodies diluted with 5% skim milk/TBST were hybridized and incubated for 2 h at room temperature. The secondary antibodies were anti-rabbit IgG (ab97051, Abcam) or anti-mouse IgG (ab97023, Abcam). The PVDF membrane was then washed with TBST three times for 10 min each and the antibodies were visualized using a chemiluminescence detection kit (Merck Millipore).

### RNA-protein pull-down assay

This assay consists with following steps: in vitro transcription, lithium chloride precipitation, RNA 3’ end desthiobiotinylation, protein precipitation, and pull-down. In vitro transcription was performed using a MEGAscript^®^ SP6 Transcription Kit (Thermo Fisher Scientific). First, the SP6 promoter-*SNHG1* plasmid was linearized through NotI digestion at 37 °C for 1.5 h. For the following in vitro transcription, NTPs, 10x reaction buffer, and enzyme mix were added to 1000 ng of the linear plasmids and the mixture was incubated at 37 °C for 4 h. To remove unincorporated proteins and nucleotides, lithium chloride precipitation was performed. The *in vitro–*synthesized *SNHG1* transcript RNA concentration was determined by NanoDrop ND-1000 (Thermo Fisher Scientific). We then calculated the 50 pmol of *in vitro–*synthesized *SNHG1* transcripts and labeled the 3’-end of the *SNHG1* RNA with a biotin marker using a Pierce^TM^ RNA 3’ End Desthiobiotinylation Kit (Thermo Fisher Scientific), according to the manufacturer’s instructions. We collected SK-N-BE(2)C cell lysate with Pierce^®^ IP Lysis Buffer containing a protease inhibitor cocktail (BioShop, Burlington, Canada), tyrosine phosphatase inhibitor cocktail, and serine/threonine phosphatase inhibitor cocktail (Bionovas, Toronto, Canada). The concentration of protein was determined using a Pierce BCA Protein Assay Kit (Thermo Fisher Scientific). After we incubated 200 µg of protein and biotin-labeled *SNHG1* transcripts, we precipitated *SNHG1*-interacting partners using a Pierce^TM^ Magnetic RNA-Protein Pull-Down Kit (Thermo Fisher Scientific). The precipitated *SNHG1*-interacting proteins were then analyzed by western blot. Streptavidin magnetic beads alone served as a negative control.

### RNA immunoprecipitation (RIP)

A RIP assay was performed using a Magna RIP^TM^ RNA-Binding Protein Immunoprecipitation Kit (Merck Millipore). The antibodies used for immunoprecipitation were HDAC1 (Abcam) or HDAC2 (Abcam). We then performed RT-PCR using the primers as follows: *SNHG1*-3’-ter-Forward-(RT) ACA GCA GTT GAG GGT TTG CT, *SNHG1*-3’-ter-Reverse-(RT) ACA GTG CCT GAG TTT GGG TT, *SNHG1*-5’-ter-Forward-(RT) GCC CAC AAG AGC TTA CTG GT, *SNHG1*-5’-ter-Reverse-(RT) TCC TCA AAC TCC TCT TGG GC, *SNHG1*-Full-off-Forward-(RT) CTC ATT TTT CTA CTG CTC G, and *SNHG1*-Full-off-Reverse-(RT) AAA CAG GAC TAT GTA ATC AAT. The PCR program was as follows: initial denaturation at 98 °C for 3 min, 30 cycles of denaturation at 98 °C for 30 s, annealing at 65 °C for 30 s, extension at 72 °C for 40 s, and a final extension at 72 °C for 10 min. All PCR products were analyzed by 1.5% agarose gel electrophoresis at 110 V for 30 min.

### RNA-seq and data analysis

Sequencing libraries were prepared using a TruSeq Stranded mRNA Preparation Kit (Illumina, San Diego, CA, USA) according to the manufacturer’s instructions and were sequenced by the Illumina NovaSeq 6000 system (performed by Genomics BioSci. & Tech., Taipei, Taiwan). Read quality was evaluated by FastQC and adaptor sequences were trimmed using cutadapt. Qualified reads were aligned to the human reference genome GRCh38 using STAR (v. 2.7.2) [15] and read counts for individual genes annotated based on GENCODE (v. 28) were subsequently determined using featureCounts [16]. Differential expression analysis was performed using limma [17] with TMM (trimmed mean of *M*-values) normalization. For pre-ranked GSEA, a ranking metric was calculated for each gene as *R* = sign(log_2_FC)* – log_10_(*p*-value), where both log_2_FC and *p*-value were determined by limma. Pre-ranked GSEA was performed using a curated collection of gene sets from MSigDB. The significant gene sets were constructed as an enrichment map [18] using an in-house script and visualized using Cytoscape.

### Assay for transposase-accessible chromatin sequencing and data analysis

ATAC-seq was performed as previously described [19]. We seeded sgSNHG1 and sgCtrl SK-N-BE(2)C cells in 10 cm dishes that were incubated for 3 days. We then centrifuged 5 × 10^3^ cells at 500 × *g* for 5 min. Cells were then washed twice with cold PBS and centrifuged at 500 × *g* for 5 min. Cells were then trypsinized using a cold lysis buffer (10 mM Tris-HCl, pH 7.4, 10 mM NaCl, 3 mM MgCl_2_ and 0.1% IGEPAL CA-630). Following lysis, nuclei were centrifuged at 500 × *g* for 10 min. To avoid losing cells during the experiment preparation, we used a fixed-angle centrifuge. After nuclei preparation, a Tn5 transposition reaction was performed using a Nextera Kit (Illumina), according to the manufacturer’s protocol. Directly following transposition, DNA was purified using a MinElute Kit (QIAGEN, Hilden, Germany). Following purification, library amplification was performed using a NEBNext High-Fidelity 2x PCR Master Mix (New England Biolabs, Ipswich, MA, USA) with SYBR Green I dye (Invitrogen, Waltham, MA, USA) and Nextera PCR primers Ad1 and Ad2. The amplified libraries were purified using a PCR purification kit (QIAGEN) and quality control was performed using the 2200 Tapestation (Agilent, Santa Clara, CA, USA). Libraries were sequenced with Illumina NextSeq 500 paired-end, resulting in 75-bp paired-end reads (Genomics BioSci. & Tech.). Two independent ATAC-seq experiments were performed for each cell line.

Adaptor sequences were removed using cutadapt and clean reads were aligned to the human genome GRCh38 using Burrows-Wheeler Alignment tool (BWA). The duplicated reads were marked by GATK-Picard. Aligned reads that were improperly paired, had multiple hits, mapped to mitochondrial genome or ENCODE blacklisted regions, or were duplicates were removed from further analysis and the position of aligned reads were shifted +4 and –5 bp for the positive and negative strands using alignmentSieve from deepTools. Aligned reads from two independent replicates were combined for peak calling. Peaks were called using MACS2 with parameters -f BAMPE –keep-dup all and annotated by ChIPSeeker [20]. Read counts of peaks for each replicate were calculated using bedtools and converted into count per million (CPM) for further analysis.

### Chromatin-immunoprecipitation sequencing and data analysis

ChIP-seq was performed as previously described [21]. The antibody H3K27ac (ab4729, Abcam) was used for ChIP. For each ChIP experiment, 10 μg of antibody was added to 3 mL of sonicated nuclear extract. Cross-linking was reversed, and DNA was purified using the Qiagen PCR purification kit (Qiagen). The DNA was used to generate sequencing libraries according to the manufacturer’s procedure (Life Technologies). Sequencing was performed with Illumina NextSeq 500, resulting in 75-bp paired-end reads (Genomics BioSci. & Tech., Taiwan). Two independent ChIP-seq experiments were performed for each cell line.

After removing adaptor sequences and low-quality reads, reads were aligned to the human genome GRCh38 using BWA. Aligned reads from two independent replicates were combined for peak calling. Peak calling was performed by MACS2 with parameters -f BAMPE -p 0.000001 –normal –extendsize 200 –keep-dup all. Peaks were annotated by ChIPSeeker [20]. Read counts of peaks for each replicate were calculated using bedtools and converted into count per million (CPM) for further analysis. Super-enhancers were identified using the ROSE algorithm [22] with some modifications.

### CCLE data analysis

The processed RNA-seq data (RSEM_TPM) were obtained from CCLE (Cancer Cell Line Encyclopedia) project website (on Sep. 28th, 2018), and neuroblastoma cell lines were extracted for further analysis according to the annotation file.

### Statistical analysis

All results are displayed as means ± SD of biological replicates. The sample size (*n*) for each experiment is indicated in the figure legends. Differences between the two groups were determined using Student’s unpaired two-tailed *t*-test. A *p*-value < 0.05 was considered statistically significant, and the significance is shown in the figures (*p < 0.05, **p < 0.01, ***p < 0.001). Statistical analyses and plots for RNA-seq, ATAC-seq and ChIP-seq were conducted using R3.5.2 (http://www.r-project.org/).

## Results

### Depletion of *SNHG1* suppresses neuroblastoma proliferation

**Fig. 1:**
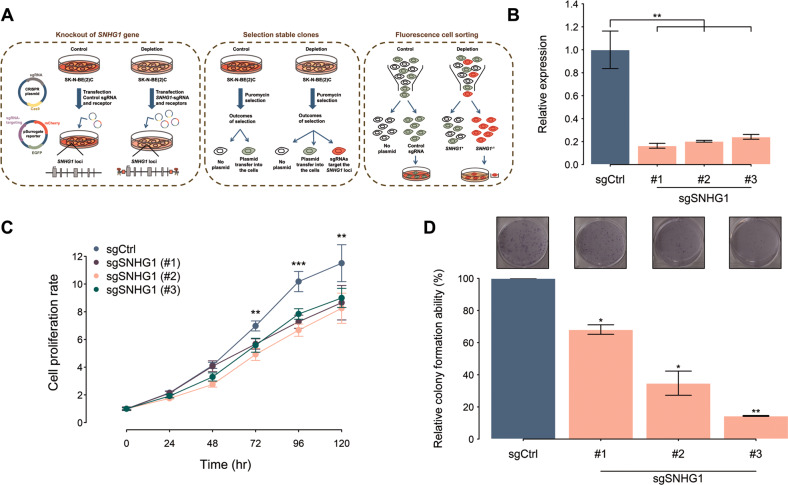
Depletion of *SNHG1* suppressed the growth of neuroblastoma cells. **A** The experimental workflow using the CRISPR/Cas9 system to create *SNHG1*-depletion neuroblastoma cells. The CRISPR plasmid contains the Cas9 protein sequence and the single guide RNA (sgRNA). The pSurrogate reporter plasmids are designed to check the efficiency of the CRISPR/Cas9 system. To deplete *SNHG1* expression, the CRISPR plasmid containing the Cas9 protein and sgRNA sequences and the reporter plasmid were co-transfected into neuroblastoma cells from the SK-N-BE(2)C cell line (left). To select stable clones showing *SNHG1*-depletion, we used a selection marker, puromycin, to choose transfected cells (middle). Fluorescence cell sorting filtered the *SNHG1*-depletion cells (right). **B** *SNHG1* expression levels measured by qRT-PCR in control sgRNA (sgCtrl) or *SNHG1*-specific sgRNAs (sgSNHG1-1, sgSNHG1-2, and sgSNHG1-3) in SK-N-BE(2)C. The expression levels were normalized to endogenous *GAPDH*. (*n* = 3) **C** Cell proliferation analysis of control sgRNA (sgCtrl) or *SNHG1*-specific sgRNAs (sgSNHG1-1, sgSNHG1-2, and sgSNHG1-3) in SK-N-BE(2)C by MTS assay. The cell numbers were measured every 24 h. (*n* = 3) **D** The colony-forming ability of control sgRNA (sgCtrl) or *SNHG1*-specific sgRNAs (sgSNHG1-1, sgSNHG1-2, and sgSNHG1-3) in SK-N-BE(2)C. SK-N-BE(2)C cells transfected with control sgRNA (sgCtrl) or *SNHG1*-specific sgRNAs (sgSNHG1-1, sgSNHG1-2, and sgSNHG1-3) were incubated for 10 days to assess colony formation. Colonies were then fixed with methanol and stained with crystal violet (upper). Colony numbers were quantified by counting (lower). (*n* = 3) Data in D are representative of three independent experiments. *p < 0.05, **p < 0.01, ***p < 0.001. See also Fig. S1.

To investigate the roles of *SNHG1* in NB, we utilized the CRISPR/Cas9 system to generate *SNHG1*-depletion stable-clone SK-N-BE(2)C cells by transfecting guide RNAs targeting *SNHG1* genomic loci (sgSNHG1) (Fig. 1A). We selected three stable clones and a clone infected by a non-targeting gRNA sequence (sgCtrl) as a negative control for measurement of *SNHG1* expression and assessment of functional effects of the *SNHG1*-depletion. In the *SNHG1*-depletion clones, *SNHG1* expression significantly decreased compared to the control clone (Fig. 1B). In addition, cell proliferation and colony formation were suppressed in SK-N-BE(2)C cells with low *SNHG1* expression (Fig. 1C, D). We also employed siRNA to silence *SNHG1* expression in SK-N-BE(2)C cells, and the *SNHG1* siRNA treatment significantly inhibited SK-N-BE(2)C cell proliferation (Fig. S1). These results highlight the oncogenic role of *SNHG1* in NB.

### Genes perturbed by *SNHG1*-depeltion are involved in various biological processes

**Fig. 2:**
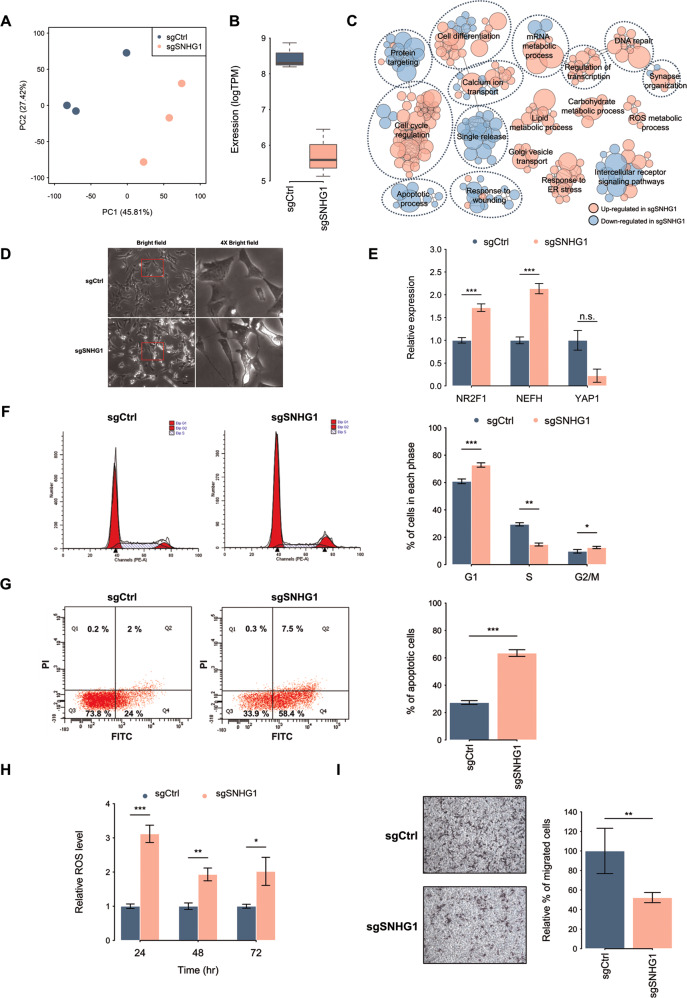
Functional analysis of *SNHG1*-depletion in neuroblastoma cells. **A** RNA-seq after control sgRNA (sgCtrl) or *SNHG1*-specific sgRNAs (sgSNHG1) transfection of SK-N-BE(2)C cells. **B** By comparing cell expression profiles with differential expression levels of *SNHG1*, we identified *SNHG1*-associated genes. **C** Analysis of biological processes correlated with *SNHG1*-associated genes in neuroblastoma cells. We used the gene set enrichment analysis (GSEA) database annotated with gene ontology (GO) terms to identify regulated genes. Each node represents an enriched GO term (corrected *p*-value < 0.05) and the size of nodes represents the *p*-value of the GO term. The color of the node indicates an up- or down-regulated gene. Functionally related GO terms are labeled. **D** The differential morphology resulting from control sgRNA (sgCtrl) or *SNHG1*-specific sgRNAs (sgSNHG1) in SK-N-BE(2)C cells (left). **E** The expression of the differentiation marker genes *NR2F1*, *NEFH*, and *YAP1* were measured by qRT-PCR. The expression levels were normalized to endogenous *GAPDH*. (*n* = 3). **F** The cell cycle analysis of SK-N-BE(2)C cells transfected with control sgRNA (sgCtrl) or *SNHG1*-specific sgRNAs (sgSNHG1) by flow cytometry (left). Propidium iodide (PI) stained the nucleus for this analysis. Quantification results are shown as a bar chart (right). (*n* = 3). **G** The cell apoptosis analysis of control sgRNA (sgCtrl) or *SNHG1*-specific sgRNAs (sgSNHG1) in SK-N-BE(2)C cells by flow cytometry. FITC-Annexin V stained phosphatidylserine (PS). PI stained the nucleus. Quantification results are shown as a bar chart (right). (*n* = 3). **H** The reactive oxygen species (ROS) analysis of control sgRNA (sgCtrl) or *SNHG1*-specific sgRNAs (sgSNHG1) in SK-N-BE(2)C cells by flow cytometry. Total ROS were detected using an H_2_DCFDA indicator. (*n* = 3). **I** The migration analysis SK-N-BE(2)C cells transfected with control sgRNA (sgCtrl) or *SNHG1*-specific sgRNAs (sgSNHG1) by a transwell assay. The cells were stained with Giemsa stain (left) and cell numbers were quantified by counting (right). (*n* = 3) Data in **D** and **I** were representative of three independent experiments. *p < 0.05, **p < 0.01, ***p < 0.001 from *t*-test.

To clarify the functions of *SNHG1* in NB, we performed an RNA-seq analysis on control and *SNHG1*-depletion SK-N-BE(2)C cells. The principal component analysis of gene expression showed the samples were grouped based on *SNHG1* status (Fig. 2A) and *SNHG1* expression was indeed suppressed in *SNHG1*-depletion cells (Fig. 2B). Gene set enrichment analysis (GSEA) of these expression profiles revealed that the depletion of *SNHG1* affected genes involved in the apoptotic process, cell cycle regulation, reactive oxygen species (ROS) metabolic process, DNA repair, regulation of transcription, mRNA metabolic process, synapse organization, cell differentiation, and response to wounding (Fig. 2C and Table S2). Functional assays were used to further validate the effect of the depletion of *SNHG1* expression, and showed induced cell differentiation (Fig. 2D, E), G1-phase cell cycle arrest (Fig. 2F), apoptosis (Fig. 2G), and ROS accumulation (Fig. 2H), as well as suppression of cell migration in NB cells (Fig. 2I).

### *SNHG1*-depletion induces cell state changes

**Fig. 3:**
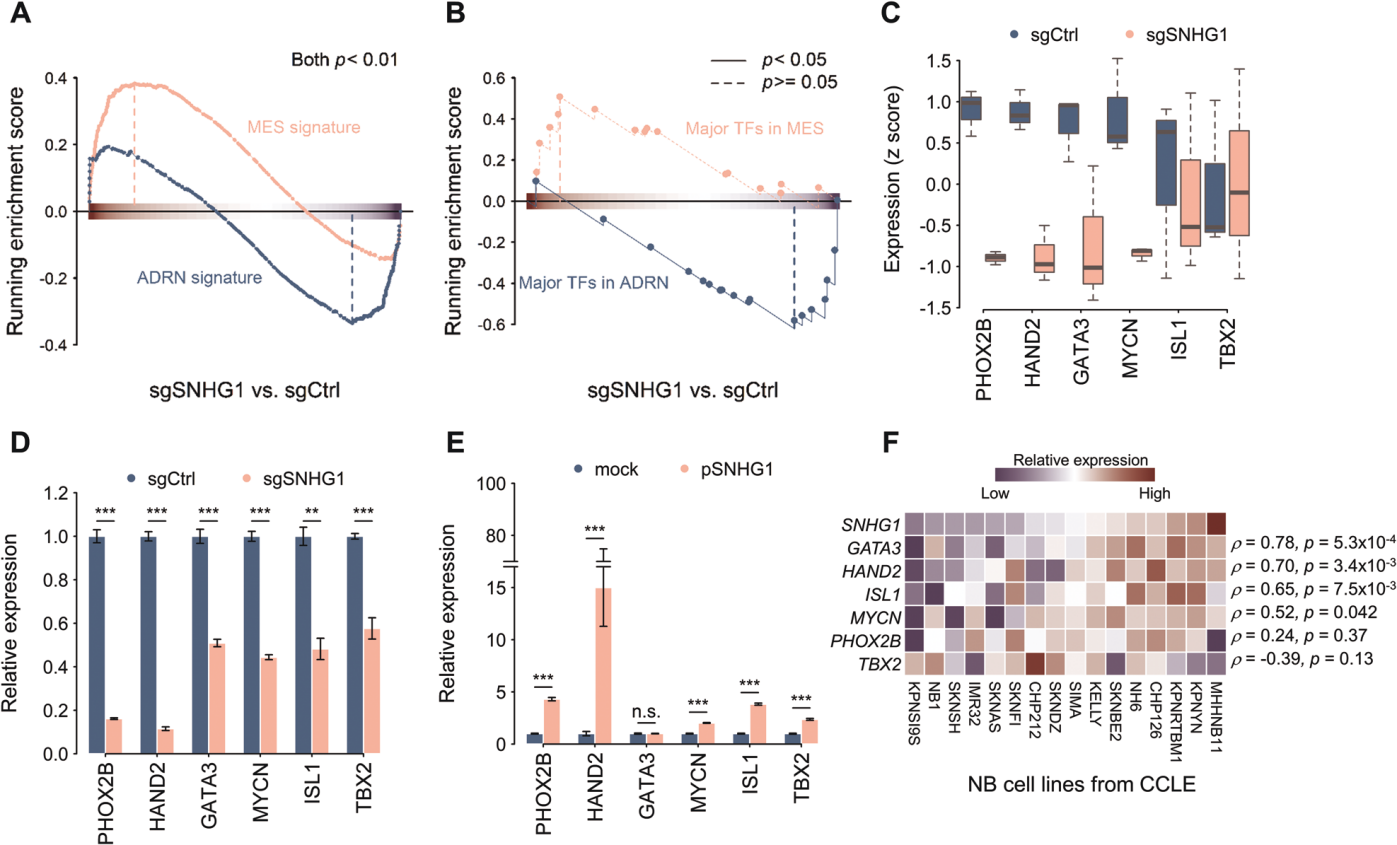
*SNHG1*-depletion decreased the expression of core regulatory circuit (CRC) transcription factors in neuroblastoma (NB) cells. **A** Mesenchymal (MES) and adrenergic (ADRN) signatures on the transcriptome profiles of *SNHG1*-depletion and control NB cells. **B** The differential expression between *SNHG1*-depletion and control NB cells were compared to the major transcriptional factors in the MES and ADRN cell states. **C** The expression of the CRC genes *PHOX2B, HAND2, GATA3, ISL1, MYCN*, and *TBX2* in the RNA-seq results. (*n* = 3). **D** CRC gene expression levels measured by qRT-PCR of control sgRNA (sgCtrl) or *SNHG1*-specific sgRNAs (sgSNHG1) in SK-N-BE(2)C cells. The expression levels were normalized to endogenous *HPRT*. (*n* = 3). **E** To dissect the relationship between CRC genes and *SNHG1*, SK-N-BE(2)C cells were transfected with a *SNHG1* overexpressing plasmid. CRC gene expression levels were measured by qRT-PCR. The expression levels were normalized to endogenous *HPRT*. (*n* = 3). **F** Gene expression heatmap of CRC genes and *SNHG1* in various NB cell lines. ρ: Spearman’s correlation coefficient. *p < 0.05, **p < 0.01, ***p < 0.001 from *t*-test.

NB cells can be classified into two cell states based on gene expression and enhancer profiles: mesenchymal (MES) and adrenergic (ADRN) cell states [23, 24]. ADRN cells are committed, but MES cells harbor migratory neural crest properties. Most *MYCN*-amplified NB cells are in an ADRN state, including SK-N-BE(2)C [24]. We investigated the MES and ADRN signatures obtained from previous work [24] on the transcriptome profile of *SNHG1*-depletion and control NB cells. We found gene expression associated with ADRN was majorly suppressed, whereas MES-associated genes were up-regulated, suggesting the cell state changes due to a loss of *SNHG1* function (Fig. 3A, B). We further examined the expression of core regulatory circuitry (CRC) members (*MYCN*, *HAND2*, *ISL1*, *PHOX2B*, *GATA3*, and *TBX2*), which are required for regulating cell growth and survival in NB [21]. Here, expression levels of these transcription factors decreased in the *SNHG1*-depletion cells, as observed in the RNA-seq data and validated by qRT-PCR (Fig. 3C, D). Conversely, we overexpressed *SNHG1* in SK-N-BE(2)C cells and the expression levels of CRC members were rescued (Fig. 3E). In addition, we examined the expression correlation of *SNHG1* to CRC transcription factors in other NB cell lines. Most of these CRC members showed good expression correlation to *SNHG1*, except for TBX2 (Fig. 3F and Table S3). We also evaluated the expression of CRC genes under the silence of *SNHG1* in two ADRN cells (SK-N-DZ and SH-SY5Y) and one MES cell (SK-N-AS) [21, 23]. The qRT-PCR assays revealed that most CRC genes were decreased in the *SNHG1*-knockdown ADRN cells compared to the control cells (Fig. S2A and S2B), but no significant differences were observed in *SNHG1*-knockdown MES cells (Fig. S2C). As a result, we speculated that *SNHG1* may play a critical role in the maintenance of NB cell fate by involving in the core regulatory circuitry, especially for the ADRN-like NB cells.

**Fig. 4:**
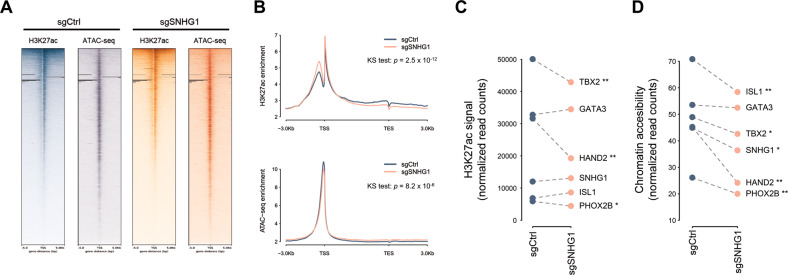
Chromatin state in cells with high and low *SNHG1* expression. **A** Heatmaps of ChIP-seq and ATAC-seq tag densities in control (sgCtrl) and *SNHG1*-depletion (sgSHNG1) cells. Data are shown in a +/− 5 kb region. **B** ChIP-seq and ATAC-seq average profiles in control (sgCtrl) and *SNHG1*-depletion (sgSHNG1) cells across H3K27ac regions. KS: Kolmogorov–Smirnov test. **C** The acetylation levels of H3K27 in CRC transcription factors (*n* = 2). **D** The chromatin states of CRC transcription factors (*n* = 2). *p < 0.05, ** *p* < 0.01 from *t*-test.

As these transcription factors, regardless of ADRN/MES cell states or CRC affiliation, have the marks of super-enhancers [21, 23, 24], we hypothesized *SNHG1* regulates NB cell fate via the formation of super-enhancers. To test this hypothesis, we performed ChIP-seq with the antibody recognizing H3K27ac, a well-recognized marker for active enhancers, and ATAC-seq in *SNHG1*-depletion and control SK-N-BE(2)C cells (Fig. 4A). Overall, the distribution of H3K27ac intensities and chromatin accessibility in *SNHG1*-depletion cells differed from control cells (*p* = 2.5 × 10^−12^ and 8.2 × 10^−5^; Kolmogorov–Smirnov test) (Fig. 4B). The H3K27ac signal and chromatin accessibility of the six CRC transcription factors decreased in *SNHG1*-depletion cells although some genes did not achieve statistical significance (Fig. 4C, D), consistent with the observations from RNA-seq and qRT-PCR (Fig. 3C, D). These findings suggest depletion of *SNHG1* dramatically changes the chromatin states of NB cells.

### *SNHG1* maintains chromatin state via HDAC1/2

**Fig. 5:**
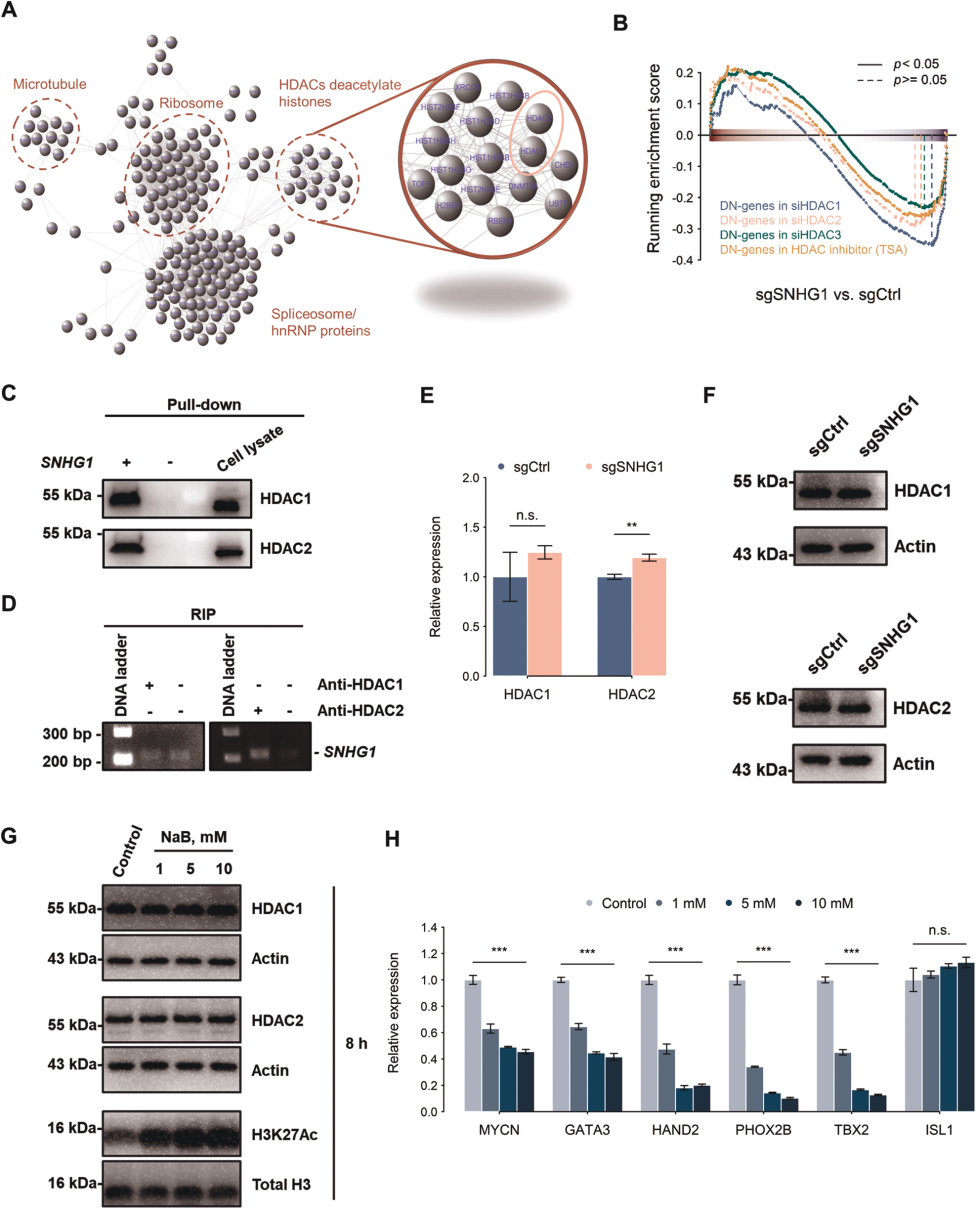
HDAC1/2 interacts with *SNHG1*. **A** Functional association network of *SNHG1*-interacting proteins constructed by STRING database. **B** The gene expression signature from cells with HDAC inhibition (TSA treatment or siHDAC) was associated with *SNHG1* depletion (sgSNHG1). **C** RNA-protein pull-down assay to detect HDAC1/2 interaction with *SNHG1* via western blot. **D** RNA immunoprecipitation (RNA-IP) validated the HDAC1/2 interaction with *SNHG1*. **E**, **F** The RNA level and protein level of HDAC1/2 were measured by qRT-PCR (E) (*n* = 3) and western blot (**F**) in control sgRNA (sgCtrl) or *SNHG1*-specific sgRNA (sgSNHG1) in SK-N-BE(2)C cells. The expression levels were normalized to endogenous *GAPDH* or actin. **G** The protein level of HDAC1/2 and acetyl-K27 of histone H3 in the sodium butyrate (NaB)-treated cells. The expression levels were normalized to endogenous actin or histone H3. **H** CRC gene expression levels measured by qRT-PCR after DMSO (control) or NaB treatment (1, 5, and 10 mM) in SK-N-BE(2)C cells. The expression levels were normalized to endogenous *HPRT*. Data in **F** and **G** were representative of three independent experiments. *p < 0.05, **p < 0.01, ***p < 0.001 from *t*-test.

We explored how *SNHG1* regulates chromatin state in NB cells. In our previous work, we used a pull-down assay coupled with mass spectrometry to identify proteins interacting with *SNHG1* in SK-N-BE(2)C cells [4]. Among the *SNHG1-*interacting proteins, a group of proteins were related to histone modification, such as HDAC1 and HDAC2 (Fig. 5A). In addition, the genes responding to the *SNHG1* depletion were associated with HDAC-responsive genes (Fig. 5B). We further verified the *SNHG1*-HDAC1/2 interaction using pull-down assays and RNA immunoprecipitation (RIP) (Fig. 5C, D; Original western blots Fig. 1A). However, *SNHG1* depletion did not affect the expression or stability of HDAC1 and HDAC2 (Fig. 5E, F; Original western blots Fig. 1B). To investigate the role of *SNHG1* and HDAC1/2 interactions, we treated SK-N-BE(2)C cells with sodium butyrate (NaB), an HDAC inhibitor. Although the total intensity of H2K27ac increased following NaB treatment, expression of CRC members was down-regulated with NaB treatment, except for *ISL1* (Fig. 5G, H; Original western blots Fig. 1C), which was consistent with *SNHG1* depletion. These results suggest *SNHG1* could serve as a positive regulator modulating chromatin state via interactions with HDAC1/2.

## Discussion

A number of studies have demonstrated that *SNHG1* serves as an oncogene in various cancer types [12]. We previously found *SNHG1* was significantly correlated with *MYCN* status and expression as well as associated with high-risk NB processes [4]. Still, the oncogenic or tumor-suppressor role of *SNHG1* in NB remains unclear. To clarify the role of *SNHG1* in NB, we adopted the CRISPR/Cas9 system to generate an *SNHG1*-depletion cell. As the functional regions or domains of *SNHG1* are unknown, we designed two single guide RNAs (sgRNAs) that target two different regions around the *SNHG1* genomic locus. Although this approach was unable to completely knock-out *SNHG1*, the expression of *SNHG1* was significantly repressed. The functional assays further demonstrated the cell growth and proliferation abilities of *SNHG1*-depletion cells were decreased. These results revealed not only the essentiality of *SNHG1* in NB growth, but also the application of this cell model for investigating the molecular mechanisms of *SNHG1*. Transcriptomics analysis of *SNHG1*-depletion cells with corresponding functional assays demonstrated the suppression of *SNHG1* affects various biological processes in NB cells, consistent with previous works [12].

Heterogeneity of NB cell identity has been identified and can be mainly classified into mesenchymal-type (MES) and adrenergic-type (ADRN) cells based on the expression of the transcriptional circuitries [24]. Distinct cell types can be interconverted by altering their transcriptional states [23, 24] and impact NB tumor development, progression, and relapse [25]. The SK-N-BE(2)C cell line belongs to the ADRN subtype of NB, and the transcriptional CRC, composed of *MYCN, HAND2, ISL1, PHOX2B, GATA3*, and *TBX2*, enables to maintain the cell state of the ADRN subtype of NB [21]. Although we demonstrated the mRNA expression of all CRC members correlate with *SNHG1* expression in NB cells, the enhancer activities (marked by H3K27ac level) of CRC members were not significantly changed in *SNHG1*-depletion cells, and *SNHG1* does not interact with CRC members (data not shown). Current results suggest *SNHG1* is a critical coregulator but not a member of the ADRN neuroblastoma CRC.

**Fig. 6:**
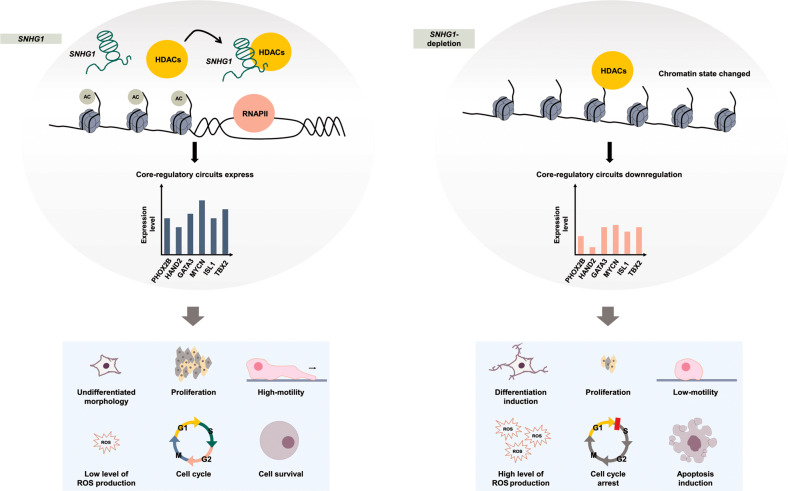
Proposed mechanism of *SNHG1* mediation in *MYCN*-amplified neuroblastoma cells. First, we examined how *SNHG1* interacted with HDAC complexes, which regulate chromatin remodeling and cancer progression. Second, we validated relationships between core regulatory circuit (CRC) genes and *SNHG1*. Together, CRC genes and *SNHG1* maintained a ADRN cell state subtype in SK-N-BE(2)C neuroblastoma cells. The depletion of those regulators lead to several biological function changes, such as cell proliferation inhibition, cell cycle arrest, a decrease in cell migration ability, induction of cell differentiation, ROS production accumulation, and an increase in apoptosis.

*SNHG1* interacting with proteins, such as hnRNPL [26], EZH2 [27], and MART3 [4], to modulate tumor progression have been reported in various cancer types. Our study shows that *SNHG1* interacts with HDAC1/2 to coordinately affect the expression of CRC members. LncRNAs modulating the epigenetic functions of HDAC has been reported. For instance, lncRNA *ANRIL* has been reported to act as a molecular scaffold to increase the formation of WDR5 and HDAC3 complex, which promotes genotypic alteration through histone modification in the vascular smooth muscle cells [28]. To further evaluate the novel epigenetic regulatory mechanism *SNHG1* is involved in, the binding regions of *SNHG1*-HDAC1/2 complex should be determined. Also, the association between the expression of *SNHG1* and the binding affinities of this histone modifier complex may provide more feasible information to deeply understand the role of SNHG1 in the phenotype switch. Notably, genes in response to the suppression of *SNHG1* are similar to that response to both HDAC silencing and the HDAC inhibitor, trichostatin A (TSA), treatment. HDACs remove lysine acetylation during chromatin condensation to decrease the expression of target genes [29], which are involved in various biological processes, including regulation of cell proliferation, cell cycle, and apoptosis [30], similar to the effects resulting from *SNHG1* depletion. Inhibition of HDAC activity has been considered a potential therapeutic strategy for anticancer treatment [31, 32]. Therefore, SNHG1 may interplay with HDAC1/2 and coordinately mediate the transition of neuroblastoma cell fate. Blockade of the *SNHG1*-HDAC interaction may be a potential target for cancer therapy in NB. However, the effect of *SNHG1*- HDAC1/2 complex on the HDAC1/2 activity and the epigenetic changes in the promoter regions of CRC members should be further investigated. Our findings demonstrate a crucial role for *SNHG1* in the regulation of chromatin state that maintains NB features. In summary, this study highlights the function of the lncRNA S*NHG1* in NB (Fig. 6).

## Supplementary information

Supplementary information

Table S1

Table S2

Table S3

A reproducibility checklist

## Data Availability

Raw data of RNA-seq, ChIP-seq, and ATAC-seq are available through GEO with accession number GSE118333 and GSE202830.
