# Supplementary material for: LncRNA SNHG1 regulates neuroblastoma cell fate via interactions with HDAC1/2
Source: Cell Death Dis. 2022 Sep 21;13(9):809. doi: 10.1038/s41419-022-05256-z (PMC9492769; doi:10.1038/s41419-022-05256-z)
Supplement: Supplementary file 1 — Supplementary information [file 41419_2022_5256_MOESM1_ESM.pdf]

Supplementary Methods

SNHG1 knockdown by siRNA

Cells were seeded into the 6 well plates and incubated for 24 h. The DsiRNAs targeting SNHG1 (Integrated DNA Technologies, Coralville, IA, USA) were transfected by jetPRIME transfection reagent (Polyplus, Illkirch, France) according to the user's manual. Scrambled DsiRNA was used as a negative control.

Supplementary Figures

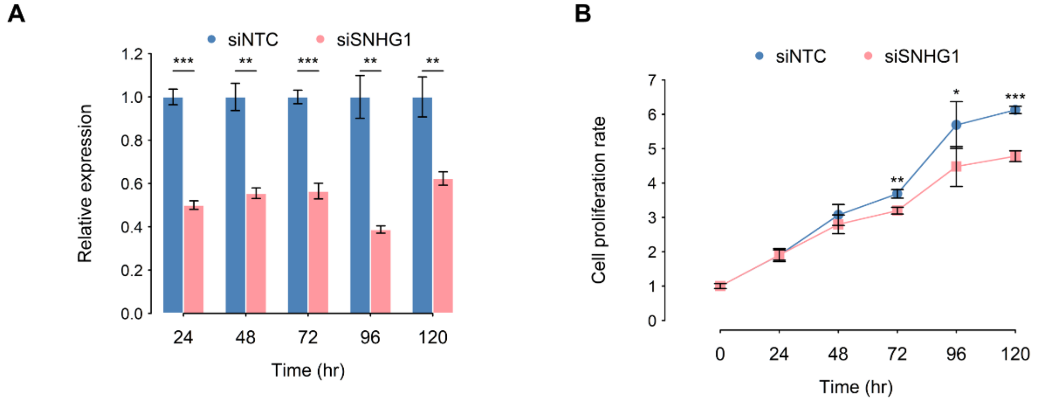

Figure S1. CRC gene expression levels and cell viability of SK-N-BE(2)C cells after infection with siNTC or siSNHG1. (A) qRT-PRC was used to measure the expression levels of CRC genes. The expression levels were normalized to endogenous *HPRT* (n=3) (B) Cell proliferation analysis of control (siNTC) or SNHG1-knockdown cells (siSNHG1) (n = 3). The statistical test was performed by Student's t-test (* $p < 0.05$, ** $p < 0.1$, and *** $p < 0.001$).

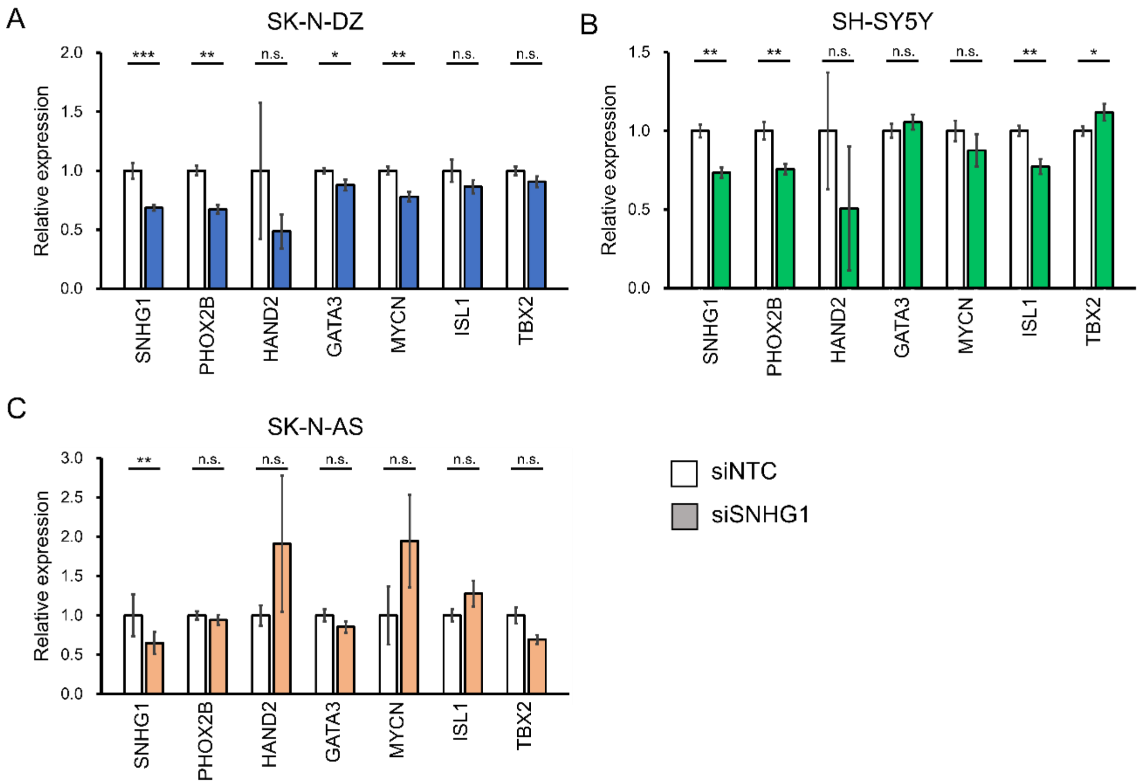

Figure S2. CRC gene expression levels in SK-N-DZ (A), SH-SY5Y (B), and SK-N-AS (C) cell lines transfected with siNTC (white) or siSNHG1 (filled color). The expression levels were measured by qRT-PCR and normalized to endogenous *HPRT* (n=3). The statistical test was performed by Student's t-test (* $p < 0.05$, ** $p < 0.1$, *** $p < 0.001$, and n.s. $p >= 0.05$).

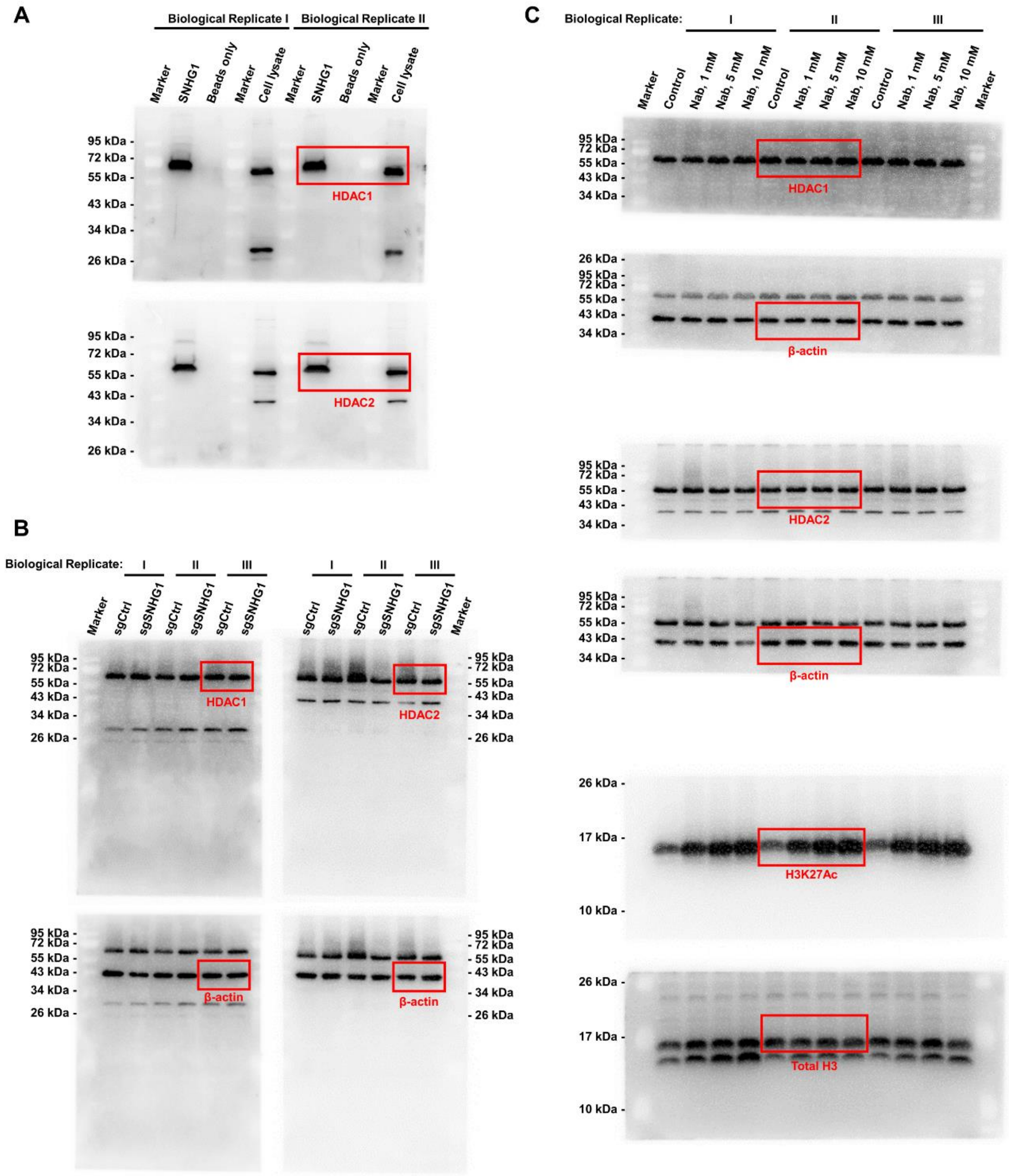

Figure S3. Full gel images of Western blotting analysis corresponding to Figure 5C (A), Figure 5F (B) and Figure 5G (C).
